# Supplementary material for: Gender and cultural aspects of brucellosis transmission and management in Nakasongola cattle corridor in Uganda
Source: PLoS One. 2025 Apr 24;20(4):e0320364. doi: 10.1371/journal.pone.0320364 (PMC12021251; doi:10.1371/journal.pone.0320364)
Supplement: S1 File — (DOCX) [file pone.0320364.s001.docx]

Supplementary file 1: Interview guide

## Focus Group Discussion Guide

**Introduction**

With the need to control and manage brucellosis infections in the country, a study has been initiated on the **Understanding the social perspectives of brucellosis management.**

The intended study will be used for academic purposes and consequently into designing policy

briefs that will inform policy. Participation in this study is voluntary and information will be treated with the utmost confidentiality

**Background information**

| Focus Group Identification number: | | | | |  | | | |
| --- | --- | --- | --- | --- | --- | --- | --- | --- |
| Type of Group (e.g., age, sex, socio-economic status, role in the community): | | | | |  | | | |
| Date: | | | | |  | | | |
| Time of focus group: | | | | |  | | | |
| Location: | | | | |  | | | |
| Description of place where discussion took place: | | | | |  | | | |
| Interviewer’s/moderator’s  names: | | | | |  | | | |
| Recorder’s name: | | | | |  | | | |
| Start/finish time: | | | | |  | | | |
| Participants: | | | | | | | | |
|  | Pseudonym or ID number | Sex | Age | Education | Occupation | Marital status | Religious denomination | Tribe/ ethnic group |
| 1 |  |  |  |  |  |  |  |  |
| 2 |  |  |  |  |  |  |  |  |
| 3 |  |  |  |  |  |  |  |  |
| 4 |  |  |  |  |  |  |  |  |
| 5 |  |  |  |  |  |  |  |  |
| 6 |  |  |  |  |  |  |  |  |
| 7 |  |  |  |  |  |  |  |  |
| 8 |  |  |  |  |  |  |  |  |

**SECTION 1. PATTERNS OF BRUCELLOSIS PREVALENCE**

1.ANIMAL- HUMAN INTERRACTION, SOCIO DEMOGRAPY AND EXPOSURE (THIS TABLE IS TESTING RESPONDENTS’ KNOWLEDGE OF THE DISEASE AND HOW IT TRANSLATES INTO PRACTICES)

1(a) Do you have any livestock at your home

2. Who looks after the livestock and what activities

| Do you eat the meat of sick animals? |
| --- |
| Do you drink raw milk? |
| Do you live close to your animals? |
| How is arbortus handled in your farms/community |
| Where is brucellosis more common(PROBE for age, area, gender,religion, livestock owners) |

**SECTION 2: SOCIAL FACTORS THAT DRIVE TRANSMISSION OF BRUCELLOSIS**

1. Do you know about brucellosis?

2. What are Animal husbandry Practices? (Probe why)

3. Are there any rituals performed in your community that can cause transmission of brucellosis?

If yes, which rituals and why, who performs them

4. What are the social factors that drive brucellosis infection and why? (Probe for cultural, economic, structural, political)

5. Where do the animals/humans get water?

6. Who are the main stakeholders in the management of brucellosis (Probe for, how they are involved, roles played by each, group interactions (social networks)?

7. What are the structural drivers of brucellosis transmission in the community (probe for market Organisation, sources of livestock, accessibility to information, regulations, slaughtering practices, trade systems, medical checkup etc.)?

**SECTION 3: EXPERIENCES AND PRACTICES ON THE RESPONSE AND CONTROL OF BRUCELLOSIS**

1. Have there been any ever any training on brucellosis management?(PROBE, what, who, communication, benefits, improvement)

2. How do you prevent transmission of brucellosis?

3. What are the implications of the preventive measures?

4. What do you do when you get symptoms of brucellosis?

5. What interventions do you take when you present with brucellosis infections?

6. Do you get advice before administering the medication? **(if no why)** ……………………….

If yes, from who/where

7. Are there any intervention strategies on prevention and control of brucellosis in your community?

8. What interventions have been put in place to help in prevention and control of brucellosis

9. How has brucellosis affected the community?

**Key Informant Interview *G*uide**

**Background information**

| Key Informant Identification  number: | | | | |  | | | |  |
| --- | --- | --- | --- | --- | --- | --- | --- | --- | --- |
| Informant information (e.g., age,  sex, socio-economic status, role in the community): | | | | |  | | | |  |
| Date: | | | | |  | | | |  |
| Time of interview: | | | | |  | | | |  |
| Location: | | | | |  | | | |  |
| Description of place where  interview took place: | | | | |  | | | |  |
| Interviewer’s/moderator’s  names: | | | | |  | | | |  |
| Recorder’s name: | | | | |  | | | |  |
| Start/finish time: | | | | |  | | | |  |
| Participants: | | | | | | | | |  |
|  | Pseudonym  or ID  number | Sex | Age | Education | Occupation | Marital  status | Religious  denomination | Tribe/ ethnic  group |  |
| 1 |  |  |  |  |  |  |  |  |  |

1. Are socio-demographic characteristics associated with the risk of brucellosis (Probe for cases of gender, age, education, religion and why)

2. What areas/locations in the community are more vulnerable to brucellosis (Probe for agricultural/nonagricultural areas, population, grassland, pastoral)

3. In your view, what activities are done during weather variations that can increase brucellosis infection

4. What are Animal husbandry Practice? In your opinion, what livestock production systems do farmers use to keep animals in this community? ***For each livestock production system probe, which zone, which farmer (rich, poor, educated, not educated, how many, why do they prefer***

5. Are there any rituals performed in your community that can cause transmission of brucellosis?

6. What are the cultural beliefs and practices, do farmers hold about prevention of brucellosis in the community *(****Probe for local name***, ***social roles? perceived causes of cultural beliefs)***

7. Are there any changes observed in brucellosis cases both humans and animals **(*Probe for past and now, what has changed and why the changes)?***

8. What is the level of awareness among community members on the increase or decrease **of** brucellosis infection and how does it translate into practice **(*probe for behavior change, education, ingestion of infected foods? geographical distribution)***

9. Who are the main stakeholders in the management of brucellosis (Probe for, how they are involved, roles played by each, group interactions (social networks)?

10. What level of communication and collaboration is between the different actors (probe for who is involved, barriers to communication, effective communication strategies).

11. What are the structural drivers of brucellosis transmission in the community (probe for market Organisation, sources of livestock, accessibility to information, regulations, slaughtering practices, trade systems, medical checkup etc.)?

12. Are there any intervention strategies on prevention and control of brucellosis?(mention them, if any)

13. In your view, what has the community benefited from these interventions?

14. What are the policy implications on brucellosis management (probe for farmers visit intervention, testing and culling, slaughter, vaccination, market trade restrictions, one health)

15. What is the impact of brucellosis on the community of Nakasongola?
