# Supplementary material for: Gender and cultural aspects of brucellosis transmission and management in Nakasongola cattle corridor in Uganda
Source: PLoS One. 2025 Apr 24;20(4):e0320364. doi: 10.1371/journal.pone.0320364 (PMC12021251; doi:10.1371/journal.pone.0320364)
Supplement: S3 File — (DOCX) [file pone.0320364.s003.docx]

Supplementary file 3: COREQ-Checklist

| **Table: Consolidated criteria for reporting qualitative studies (COREQ): 32-item checklist** | | | |
| --- | --- | --- | --- |
|  |  |  |  |
| **No Item Guide** | **Item no** | **questions/description** | **Page number** |
| **Domain 1: Research team and reflexivity**  Personal Characteristics | | | |
| Interviewer/facilitator | 1. | Which author/s conducted the interview or focus group? | 1 |
| Credentials | 2. | What were the researcher’s credentials? E.g. *PhD, MD* | 1 |
| Occupation | 3. | What was their occupation at the time of the study? | 1 |
| Gender | 4. | Was the researcher male or female? |  |
| Experience and training | 5. | What experience or training did the researcher have? | 4 |
| Relationship with participants | | | |
| Relationship established | 6. | Was a relationship established prior to study commencement? |  |
| Participant knowledge of the interviewer | 7. | What did the participants know about the researcher? e.g. *personal goals, reasons for doing the*  *research* |  |
| Interviewer characteristics | 8. | What characteristics were reported about the interviewer/facilitator? e.g. *Bias, assumptions, reasons and interests in the research topic* | 4 |
| **Domain 2: study design**  Theoretical framework | | | |
| Methodological orientation and  Theory | 9 | What methodological orientation was stated to underpin the study? e.g. *grounded theory,*  *discourse analysis, ethnography, phenomenology, content analysis* | 4 |
| Participant selection | | | |
| Sampling | 10 | How were participants selected? e.g. *purposive, convenience, consecutive, snowball* | 4 |
| Method of approach | 11 | How were participants approached? e.g. *face-to-face, telephone, mail, email* | 4-5 |
| Sample size | 12. | How many participants were in the study? | 4-5 |
| Non-participation | 13 | How many people refused to participate or dropped out? Reasons? | N/A |
| Setting | | | |
| Setting of data collection | 14. | Where was the data collected? e.g. *home, clinic, workplace* | 5 |
| Presence of non-participants | 15. | Was anyone else present besides the participants and researchers? | No |
| Description of sample | 16. | What are the important characteristics of the sample? e.g. *demographic data, date* | 4 |
| Data collection | | | |
| Interview guide | 17. | Were questions, prompts, guides provided by the authors? Was it pilot tested? | 5 |
| Repeat interviews | 18. | Were repeat interviews carried out? If yes, how many? | N/A |
| Audio/visual recording | 19. | Did the research use audio or visual recording to collect the data? | 5-6 |
| Field notes | 20. | Were field notes made during and/or after the interview or focus group? | 4-5 |
| Duration | 21. | What was the duration of the interviews or focus group? | 4 |
| Data saturation | 22. | Was data saturation discussed? | 4 |
| Transcripts returned | 23. | Were transcripts returned to participants for comment and/or correction? | N/A |
| **Domain 3: analysis and findings**  Data analysis | | | |
| Number of data coders | 24. | How many data coders coded the data? | 4 |
| Description of the coding tree | 25. | Did authors provide a description of the coding tree? | 4-5 |
| Derivation of themes | 26. | Were themes identified in advance or derived from the data? | 4-5 |
| Software | 27. | What software, if applicable, was used to manage the data? | 5 |
| Participant checking | 28. | Did participants provide feedback on the findings? |  |
| Reporting | | | |
| Quotations presented | 29. | Were participant quotations presented to illustrate the themes / findings? Was each quotation identified? e.g. *participant number* | 9-13 |
| Data and findings consistent | 30. | Was there consistency between the data presented and the findings? | 9-15 |
| Clarity of major themes | 31. | Were major themes clearly presented in the findings? | 9-13 |
| Clarity of minor themes | 32. | Is there a description of diverse cases or discussion of minor themes? | 13-15 |
