# Supplementary material for: Gender and cultural aspects of brucellosis transmission and management in Nakasongola cattle corridor in Uganda
Source: PLoS One. 2025 Apr 24;20(4):e0320364. doi: 10.1371/journal.pone.0320364 (PMC12021251; doi:10.1371/journal.pone.0320364)
Supplement: Attachment — Submitted filename: REVIEW~1.DOC [file pone.0320364.s004.DOC]

Gender and Cultural Aspects of Brucellosis Transmission and Management in Nakasongola Cattle Corridor in Uganda

Abstract: How data was analyzed was not reflected in the methods.

Results. It is common knowledge that practices such as consumption of raw milk, assisting in births rooted in social norms aid in transmission of brucellosis. What the study found on these in the study area is not clear in the abstract.

Conclusion: The issues raised are quite critical in brucellosis transmission and control. However, how these apply in the study were not elucidated.

Affiliations 1 & 2 should be well separated.

Introduction: The authors were unable to elucidate clearly the importance of ‘gender’ in the ‘Social Ecological Model of Human Behaviour (SEMHB)’ theoretical framework in creating a … that resulted in undertaking the study. While ‘vulnerability and social construction’ may be clear to me, the gender dynamics is not. The authors may wish to add a few lines improve the understanding of everyone.

Lines 66 and 67: ‘*Brucella melitensis*’ should be correctly spelt. ‘*Brucella*’ in the three different species mentioned should start with upper cases.

Line 77: ‘…existing literature have…’ There were more than one literature named.

Lines 127 – 129: Please, recast the sentence.

Line 146: ‘40-45 minutes’ should be written with spaces ’40 - 45 minutes’

Line 269: Should read ‘*Milk keeps us warm, healthy and not…’* Although quoting a source, the whole excerpt was rephrased and therefore should be consistent. Same as line 282 : ‘*until its’ should be ‘until it is’*

334: ‘.. a lot effort ..’ should read ‘.. a lot of effort ..’

361: ‘..*a mulalo women..’ ‘..a mulalo woman..’*

Discussion

429 ; ‘This conquers’ should be written ‘This concurs’

443: Kansime et al, 2014 (32) should be written in conformity with other citations in the manuscript: Kansime et al. (32)

497: ‘,,understand the intended question’ to read ‘...understood the intended question’

Conclusion: The conclusion should reflect the findings of the work. The statements in the conclusions sound general and did not reflect their connection to the work.
