# Supplementary material for: Gender and cultural aspects of brucellosis transmission and management in Nakasongola cattle corridor in Uganda
Source: PLoS One. 2025 Apr 24;20(4):e0320364. doi: 10.1371/journal.pone.0320364 (PMC12021251; doi:10.1371/journal.pone.0320364)
Supplement: Attachment — Submitted filename: Response to Editors.docx [file pone.0320364.s007.docx]

Makerere University,

College of Humanities and Social Sciences

8th November 2024

To: The Editor,

PLOS ONE

RE. RESPONSE TO EDITOR

We express our sincere gratitude to the editor for granting us an opportunity to address the data availability requirements for our manuscript, titled “**Gender and Cultural Aspects of Brucellosis Transmission and Management in Nakasongola Cattle Corridor in Uganda**”.

We are committed to ensuring compliance with PLOS’s Data Policy and long-term access to our study data. We have made the necessary arrangements as follows:

1. Data Sharing via Supplementary Files: The minimal data required for replication of our results will be made publicly accessible by including it as supplementary files attached to the manuscript submission.

Kind regards,

Corresponding Author:

Christine Tricia Kulabako

Email: kulabakokristin1@gmail.com

Phone: +256779688004
