# Supplementary material for: Gender and cultural aspects of brucellosis transmission and management in Nakasongola cattle corridor in Uganda
Source: PLoS One. 2025 Apr 24;20(4):e0320364. doi: 10.1371/journal.pone.0320364 (PMC12021251; doi:10.1371/journal.pone.0320364)
Supplement: Attachment — Submitted filename: Comments.docx [file pone.0320364.s006.docx]

**Manuscript number**: PONE-D-24-16528R1

**Manuscript title**: Gender and Cultural Aspects of Brucellosis Transmission and Management in Nakasongola Cattle Corridor in Uganda

To editor,

This manuscript explores a critical public health issue that affecting human health negatively. The study highlighted on the brucellosis transmission vulnerability with gender dynamics, and sociocultural construction that can inform targeted interventions for the development to control measures and management strategies that will contribute to improve health outcomes for affected population especially the pastoralist communities in Africa.

That said, I would like to offer the following comments and suggestions:

1. **Abstract**

In line 35 and 36, the author write about the use of deductive and inductive approaches is overlapping.

2. **Introduction**

Okay.

3. **Methods**

Methods section is well written.

- However, the author need to revise about the sample size part. The authors did not explain about the justification of 4 FGDs and 15 KIIs scientifically. In addition, 1 FGD conducted with combined group but why it was not equal or shy it was combined is not explained. As the study focused on the gender dynamics, vulnerability and social construction in the management of brucellosis, the justification of sampling size and sampling strategy would be more evident.
- Additionally, the author can revise it as the study population and sampling strategies. Because, how the study participants recruited, participants rights, interview guidelines were developed did not describe. Better to revise it into separate part like sample size and sampling strategies and then data collection technique and procedures. Additionally, it would be more evident if the authors follow the COREQ.
- In line191, the author present ‘the context and interpretation……..enhanced by observation ….and field notes made during the interview…. is not mentioned in the data collection as a technique. In line 171-172, the author present that key points were brought up….and triangulated with observation which should move into data analysis.
- The triangulation issues are not only confined with the comparing data with key points (line 171-172), it would be participants, methods like discussion data with interview and/or observation. Suggesting to follow a citation.
- Line 175-179 suggesting move to the end of discussion as this writing are seemed as limitations of study.
- Data analysis part is not articulated well. How the audio data transcribed, familiar with the data, code list developed and updated are not explained? The authors started directly with the thematic analysis and then talk about the Nvivo transformation and so on.
- Additionally, how the code list entail following both deductive and inductive way? Need to explain.

4. **Results**

Okay but I would ask to include the interview number in the quotes.

5. **Discussion**

- In line 435, the authors just mentioned about SEMHB model but did not elaborated any in the discussion section. Although the findings are mostly interlinked with the model but did not discussed.
- In line 505, the author write: To address this limitation, ‘I’ developed… This is might be typo. Because in the methods section, the author addresses the term ‘We’. Need to revise one addressing style.
