# Supplementary material for: Gender and cultural aspects of brucellosis transmission and management in Nakasongola cattle corridor in Uganda
Source: PLoS One. 2025 Apr 24;20(4):e0320364. doi: 10.1371/journal.pone.0320364 (PMC12021251; doi:10.1371/journal.pone.0320364)
Supplement: Attachment — Submitted filename: Response to Reviewers.docx [file pone.0320364.s008.docx]

Makerere University,

College of Humanities and Social Sciences

24^th^ January 2025

To: The Editor,

PLOS ONE

RE. AUTHORS’ REBUTTAL LETTER

We express our sincere gratitude to the editor for granting us another opportunity to revise our manuscript entitled “**Gender and Cultural Aspects of Brucellosis Transmission and Management in Nakasongola Cattle Corridor in Uganda**”. We really appreciate the peer reviewers' hard work and insightful feedback, which has greatly improved our manuscript. We have addressed the reviewers’ comments to the best of our ability and hence, we would like to resubmit the revised manuscript. Find below in a table the author's response to the reviewers’ comments, in the response, we provide line numbers of the track changed version of the manuscript not the clean version for your reference.

Thanks.

| **No** | **Comment** | **Response** |
| --- | --- | --- |
| 1 | Abstract: In line 35 and 36, the author write about the use of deductive and inductive approaches is overlapping. | Thank you for your observation, this has been edited in line 36 |
| 2 | Introduction: Okay. | Thank you for the comment |
| 3 | **Methods**  Methods section is well written.   - However, the author need to revise about the sample size part. The authors did not explain about the justification of 4 FGDs and 15 KIIs scientifically. In addition, 1 FGD conducted with combined group but why it was not equal or shy it was combined is not explained. As the study focused on the gender dynamics, vulnerability and social construction in the management of brucellosis, the justification of sampling size and sampling strategy would be more evident. | Thank you for the insight. Scientific justification of 4FGDs and 15 KIIs has been explained. The sample size was based on participant saturation using maximum variation strategy, line 143-145.  The reason for unequal FGDs has also been included, Line 155-157 |
| 4 | Additionally, the author can revise it as the study population and sampling strategies. Because, how the study participants recruited, participants rights, interview guidelines were developed did not describe. Better to revise it into separate part like sample size and sampling strategies and then data collection technique and procedures. Additionally, it would be more evident if the authors follow the COREQ. | Thank you, this has been revised, recruitment and how interview guidelines were developed has been included, line 146-149.  168-170 and 175-180. Guidance of the COREQ has been included, Line 210-213. |
| 5 | In line191, the author present ‘the context and interpretation……..enhanced by observation ….and field notes made during the interview…. is not mentioned in the data collection as a technique. In line 171-172, the author present that key points were brought up….and triangulated with observation which should move into data analysis. | Thank you for your observation and insight. Observation has been deleted and moved to data analysis, Line 203-206 |
| 6 | The triangulation issues are not only confined with the comparing data with key points (line 171-172), it would be participants, methods like discussion data with interview and/or observation. Suggesting to follow a citation. | This has been revised to include a citation, Line 202 |
| 7 | Line 175-179 suggesting move to the end of discussion as this writing are seemed as limitations of study. | Thank you for the insight, this has been moved the discussion, ine 520-524 |
| 8 | Data analysis part is not articulated well. How the audio data transcribed, familiar with the data, code list developed and updated are not explained? The authors started directly with the thematic analysis and then talk about the Nvivo transformation and so on. | Thank you for the observation, this has been revised, Line 200-203 |
| 9 | Additionally, how the code list entail following both deductive and inductive way? Need to explain. | Revised in Line 200-201 |
| 10 | **Results**  Okay but I would ask to include the interview number in the quotes. | Thank you, interview numbers have been included on the quotes |
| 11 | **Discussion**  In line 435, the authors just mentioned about SEMHB model but did not elaborate any in the discussion section. Although the findings are mostly interlinked with the model but did not discuss. | Thank you for the insight, an elaboration of the SEMHB has been included, Line 457-465 |
| 12 | In line 505, the author writes: To address this limitation, ‘I’ developed… This is might be typo. Because in the methods section, the author addresses the term ‘We’. Need to revise one addressing style. | This has been edited, Line 523 |

Kind regards,

Corresponding Author:

Christine Tricia Kulabako

Email: kulabakokristin1@gmail.com

Phone: +256779688004
